# Martingales and the fixation time of evolutionary graphs with arbitrary dimensionality

**DOI:** 10.1098/rsos.220011

**Published:** 2022-05-11

**Authors:** Travis Monk, André van Schaik

**Affiliations:** International Centre for Neuromorphic Systems, The MARCS Institute, Western Sydney University, Sydney, Australia

**Keywords:** Moran process, stochastic process, birth–death process, evolutionary model, fixation time

## Abstract

Evolutionary graph theory (EGT) investigates the Moran birth–death process constrained by graphs. Its two principal goals are to find the fixation probability and time for some initial population of mutants on the graph. The fixation probability of graphs has received considerable attention. Less is known about the distribution of fixation time. We derive clean, exact expressions for the full conditional characteristic functions (CCFs) of a close proxy to fixation and extinction times. That proxy is the number of times that the mutant population size changes before fixation or extinction. We derive these CCFs from a product martingale that we identify for an evolutionary graph with any number of partitions. The existence of that martingale only requires that the connections between those partitions are of a certain type. Our results are the first expressions for the CCFs of any proxy to fixation time on a graph with any number of partitions. The parameter dependence of our CCFs is explicit, so we can explore how they depend on graph structure. Martingales are a powerful approach to study principal problems of EGT. Their applicability is invariant to the number of partitions in a graph, so we can study entire families of graphs simultaneously.

## 1. Introduction

Evolutionary graph theory (EGT) studies how spatial constraints affect evolutionary processes, e.g. the Moran birth–death process [1,2]. The original Moran process models two species called ‘mutants’ and ‘residents’ that reproduce and die over time until either species goes extinct [3]. The difference between the species is that mutants are chosen to reproduce with a different probability relative to residents, i.e. mutants have a ‘fitness’. On a time step, we choose one individual to reproduce and replace another individual we choose uniformly at random. The offspring of any individual can replace any other, so there are no spatial constraints on the process. EGT extends the Moran process by running it on a graph, where nodes are individuals and connections constrain which individuals may be replaced by another’s offspring [4–8]. Two principal goals of EGT are to find the probability that mutants drive the residents to extinction (i.e. the ‘fixation probability’), and the distribution of the time it took (i.e. the ‘fixation time’) [9–11]. We can study how space constrains evolutionary processes by finding the relationship between these quantities and graph structure.

The fixation probability is well known for the Moran process on various graphs [4,6–8,12–20]. Finding those graphs’ distributions of fixation and extinction time is a much harder problem [11,21–25]. We can simplify that problem by imposing some limit, e.g. large population size or weak selection, or by restricting focus to the distributions’ means [9,22,24,25]. But general, exact and tractable expressions for those distributions have not been reported for the original Moran process [10,11], let alone for its extension on graphs.

Martingales offer a different approach to this difficult problem [6,11,26–28]. A martingale is a conservation statement for certain quantities in a stochastic process. From that conservation statement, we can extract statistics about those quantities upon fixation or extinction. For example, Wald found a martingale for the sum of independent and identically distributed (i.i.d.) random variables and the number of observations in that sum [29–32]. From that martingale, he found the probability that the sum will hit one stopping barrier before another (i.e. the fixation and extinction probabilities). He also found the conditional characteristic functions (CCFs) of the number of observations required to hit one stopping barrier before another (i.e. the fixation and extinction times). Wald’s key assumption is that the random variables comprising the sum are i.i.d.

The original Moran process is very similar to Wald’s problem [11]. Say Wald’s sum is the mutant population size of the Moran process at some time. Then the random variables comprising that sum are the changes in the mutant population size over previous time steps. The number of observations in the sum is the current time of the process. Let Wald’s stopping barriers represent extinction (the sum hits 0) and fixation (the sum hits the total population size). We want to find the probability that the sum hits one barrier before the other, and how many observations are needed to do so.

Unfortunately, we cannot directly apply Wald’s analysis to the Moran process because his key assumption is not met. The changes in mutant population size over time steps are not independent of each other (i.e. the transition probabilities are not i.i.d.). But if we eliminate time steps where a mutant offspring replaces a mutant or a resident offspring replaces a resident, then Wald’s key assumption is met [11,30]. So we can apply Wald’s analysis to the Moran process if we discard time steps where the mutant population size does not change. Therefore we can find elegant expressions for the exact CCFs of the number of mutant population size changes before extinction or fixation, i.e. the CCFs of the number of ‘active steps’ [23,33]. The fixation and extinction time distributions remain open problems, but martingales yield clean, elegant and exact expressions for a close proxy to them.

We want to find conditions under which this martingale analysis can be extended to consider the Moran process on graphs. One condition is that the graph must have connections of a certain type. We can connect individuals on graphs with a variety of connection types. Undirected connections allow both mutant and resident offspring to travel in either direction along a graph edge [13,34–36]. Directed connections constrain both species’ offspring to travel in one identical direction along a graph edge [37,38]. Street connections constrain both species’ offspring to travel in opposite directions along a graph edge [26]. We call them ‘streets’ because, like traffic, offspring travels along a graph edge in opposing directions depending on the species of its parent.

For example, a graph’s partitions might represent colonies of sponges on the seabed that emit larval offspring [39]. If those partitions are connected by streets, then mutant larvae swim away from their parent in one direction and resident larvae swim away in the other direction. Perhaps the mutation causes the larval cilia to beat in reverse with respect to the residents [40]. So street connections impose an extra phenotypical discrepancy between mutants and residents in addition to fitness. That extra discrepancy impacts fixation probabilities and times, and that impact depends on the structure of the graph [26].

We will extend Wald’s martingale methodology to analyse the Moran process on graphs with any number of partitions. We will consider a *k*-partite street graph as a *k*-dimensional random walk between two stopping barriers representing fixation and extinction. We will identify a *k*-dimensional product martingale similar in form to Wald’s one-dimensional version. We will then obtain the fixation probability and the full CCFs of ‘active steps’ from that martingale [23]. Our results are the first general expressions for the full CCFs of any proxy to fixation time on a graph with more than two partitions [41]. We only require the elimination of time steps where the mutant population size does not change, and that the graph’s partitions are connected by streets. The parameter dependence of our CCFs is explicit, so it is easy to explore how they depend on graph structure. Our results highlight that martingales scale naturally with dimensionality. So we can consider a street graph with any number of partitions and the application of martingale analysis does not increase in difficulty. Since martingales can circumvent the curse of dimensionality, they can tractably analyse entire families of graphs simultaneously. This property makes martingales particularly powerful tools to study key problems of EGT.

## 2. Results

### 2.1. Problem statement and notation

For an overview of the Moran birth–death process, see [2,5]. To review an application of Wald’s martingale to the Moran process, see [11]. To review an application of martingales for fixation time CCFs on bipartite graphs, see [41].

**Figure 1.**
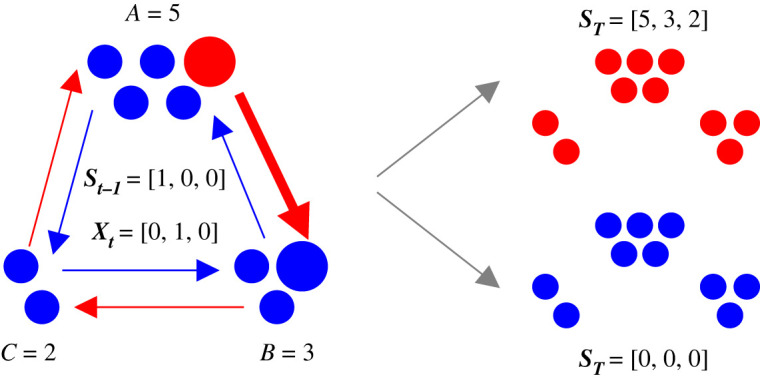
Schematic of the Moran process on a tripartite street graph and corresponding notation. The left graph is a tripartite street graph where individuals (blue and red circles) are divided into three partitions with sizes *A* = 5, *B* = 3 and *C* = 2. Street connections constrain mutants (red circles) to reproduce in one direction around the graph and residents (blue circles) in the other (red and blue arrows). In this example, mutants reproduce clockwise and residents counter-clockwise. ***S*_*t*−1_** represents the number of mutants in each partition on time step *t* − 1, and ***X*_*t*_** is the change in the mutant population size on time step *t*. On this example time step, the mutant in partition *A* replaces a resident in *B* (enlarged individuals and arrow), so the mutant population in partition *B* increases by 1. We repeat the Moran process, sequentially selecting individuals to reproduce and die, until all individuals are mutants (upper-right graph) or residents (lower-right graph). The two graphs on the right represent the two possible absorbing states of ***S***_***T***_, ***a*** = [5, 3, 2] and ***b*** = [0, 0, 0].

Figure 1 illustrates the Moran birth–death process on a tripartite street graph [26]. A population of individuals is divided into three partitions of sizes *A*, *B* and *C*, e.g. *A* = 5, *B* = 3 and *C* = 2 in figure 1. All individuals are either mutants (red circles) or residents (blue circles). There are two differences between the two species. First, mutants are chosen to reproduce on a time step with a different probability relative to the residents. This difference is quantified by a ‘fitness parameter’ *r* that is intended to model selective advantages or disadvantages [2]. Second, mutant and resident offspring reproduce in different directions around the graph. In figure 1, mutant offspring go clockwise around the graph (red arrows), and resident offspring go counter-clockwise around it (blue arrows).

The mutant population on an evolutionary graph with some integer number of partitions *k* can be considered as a *k*-dimensional random walk, where the mutant population size in each partition corresponds to one dimension of the random walk. To index the partitions, we define an alphabet a,b,…,ω with the same number of letters *k* as there are partitions in the graph. Say the capitalized letters of that alphabet A,B,…,Ω represent the number of individuals in each partition. For example, in figure 1, *k* = 3 partitions in the graph, *ω* = *c* (the last letter of our indexing alphabet), and Ω=C=2 individuals are in that partition.

We use bold letters to denote vectors. Let ***S***_***t***−**1**_ = [*S*_*a*,*t*−1_, *S*_*b*,*t*−1_, …, *S*_*ω*,*t*−1_] be a vector whose elements represent the mutant population size in each partition on time step *t* − 1. For example, ***S***_***t***−**1**_ = [1, 0, 0] for the left graph in figure 1. Let ***X***_***t***_ = [*X*_*a*,*t*_, *X*_*b*,*t*_, …, *X*_*ω*,*t*_] be the change of the mutant population size on time step *t*. Figure 1 illustrates one example time step where a mutant from partition A reproduces, and its offspring replaces a resident in B (enlarged circles and arrow, figure 1). The current state of the mutant population size is the sum of its changes over previous time steps, plus its initial state. We write $S_{T}=\sum_{\;j=1}^{T}X_{\;j}+S_{0}$, where ***S*****_0_** = [*S*_*a*,0_, *S*_*b*,0_, …, *S*_*ω*,0_] is the initial mutant population size. We continue making observations of ***X***_***t***_ and cumulatively summing them until all individuals are mutants (top right graph, figure 1) or residents (bottom right graph). The process terminates at one of these two absorbing states at some random time *T*.

EGT generally has two main goals. First, we want to find the ‘fixation probability’, i.e. the probability that the initial mutant population ***S***_0_ eventually dominants the residents. Let a=[A,B,…,Ω] (fixation) and ***b*** = [0, 0, …, 0] (extinction) represent the two possible absorbing states of the evolutionary graph. We denote the fixation probability as $\mathrm{Pr}(S_{T}=a)\equiv \alpha$ and the extinction probability as $\mathrm{Pr}(S_{T}=b)=1-\alpha$. Second, we want to find the (conditional) distributions of time steps *T* required for the graph to achieve those absorbing states, $\mathrm{Pr}{\left(T|S_{T}=a\right)}_{t=0}^{\infty }$ and $\mathrm{Pr}{\left(T|S_{T}=b\right)}_{t=0}^{\infty }$.

Those conditional time distributions are very difficult to calculate, even for the simpler fully connected Moran process [9–11]. Instead, we consider the number of times that the mutant population size changes before mutants fix or go extinct *C*_*T*_. That is, *C*_*T*_ represents the number of ‘active steps’ in the process [23]. Let $Y_{t}$ denote whether or not the mutant population size changes on time step *t*:

$$Y_{t}=1\;\mathrm{if}\;X_{t}\neq [0,\;0,\ldots ,0];\;Y_{t}=0\;\mathrm{if}\;X_{t}=[0,0,\ldots ,0].$$

Initializing *C*_0_ = 0, we can write $C_{T}=\sum_{\;j=1}^{T}Y_{j}$. Note that *C*_*T*_ depends on *T*, so we interpret ‘active steps’ as a proxy to fixation or extinction time [11].

We will identify product martingales that yield *α* and the full CCFs of *C*_*T*_ for *k*-partite street graphs.

### 2.2. Extracting absorption probabilities and conditional characteristic functions from a multidimensional martingale

Assume we have a product martingale of the form [29–32]:

2.1 $$E\left[g^{C_{t}}\prod_{i=a}^{\omega }{\;f_{i}(g)}^{S_{i,t}}|S_{t-1},C_{t-1}\right]=g^{C_{t-1}}\prod_{i=a}^{\omega }{\;f_{i}(g)}^{S_{i,t-1}},$$

where *g* is a free complex variable, and all *f*_*i*_(*g*) are functions of *g* *that are independent of* ***S*_*t*−1_**. The index *i* of the product runs over our whole alphabet *a* to *ω*, so there is one function *f*_*i*_(*g*) for each partition of the street graph. For example, the tripartite street graph will have three state-independent functions *f*_*a*_(*g*), *f*_*b*_(*g*) and *f*_*c*_(*g*). *i* also indexes the elements of $S_{t}$, i.e. the *i*th element of $S_{t}$ is the mutant population size of the *i*th partition.

The terms *C*_*t*_ and *S*_*i*,*t*_ in equation (2.1) are sums. The exponential of a sum can be expressed as a product, so we can write:

$$\begin{array}{rl} E\left[g^{C_{t}}\prod_{i=a}^{\omega }{\;f_{i}(g)}^{S_{i,t}}|S_{t-1},C_{t-1}\right] & =E\left[g^{C_{t-1}}g^{Y_{t}}\prod_{i=a}^{\omega }{\;f_{i}(g)}^{X_{i,t}}\prod_{i=a}^{\omega }{\;f_{i}(g)}^{S_{i,t-1}}|S_{t-1},C_{t-1}\right]\\ & =g^{C_{t-1}}\prod_{i=a}^{\omega }{\;f_{i}(g)}^{S_{i,t-1}}E\left[g^{Y_{t}}\prod_{i=a}^{\omega }{\;f_{i}(g)}^{X_{i,t}}|S_{t-1},C_{t-1}\right]. \end{array}$$

If we can show that the last conditional expectation equals 1, then equation (2.1) is true. We say that *g* and all *f*_*i*_(*g*) satisfying equation (2.1) define a product martingale.

We can calculate the fixation probability and CCFs of *C*_*T*_ from equation (2.1) [30]. Taking the expectation of both sides of equation (2.1):

$$E\left[g^{C_{t}}\prod_{i=a}^{\omega }{\;f_{i}(g)}^{S_{i,t}}\right]=E\left[g^{C_{t-1}}\prod_{i=a}^{\omega }{\;f_{i}(g)}^{S_{i,t-1}}\right].$$

So by induction:

$$E\left[g^{C_{t}}\prod_{i=a}^{\omega }{\;f_{i}(g)}^{S_{i,t}}\right]=E\left[g^{C_{0}}\prod_{i=a}^{\omega }{\;f_{i}(g)}^{S_{i,0}}\right]=\prod_{i=a}^{\omega }{\;f_{i}(g)}^{S_{i,0}},$$

where the last equality assumes that ***S*_0_** is known (non-random) and initializes *C*_0_ = 0. Doob’s optional stopping theorem states that a randomly stopped martingale is still a martingale [42,43]. So we can insert a random variable *T* for *t*:

$$E\left[g^{C_{T}}\prod_{i=a}^{\omega }{\;f_{i}(g)}^{S_{i,T}}\right]=\prod_{i=a}^{\omega }{\;f_{i}(g)}^{S_{i,0}}.$$

Split this expectation conditional on extinction or fixation:

$$E\left[g^{C_{T}}\prod_{i=a}^{\omega }{\;f_{i}(g)}^{S_{i,T}}|S_{T}=a\right]\alpha +E\left[g^{C_{T}}\prod_{i=a}^{\omega }{\;f_{i}(g)}^{S_{i,T}}|S_{T}=b\right](1-\alpha )=\prod_{i=a}^{\omega }{\;f_{i}(g)}^{S_{i,0}}.$$

Insert the extinction and fixation boundaries ***a*** and ***b***:

2.2 $$\left(\prod_{i=a}^{\omega }{\;f_{i}(g)}^{a_{i}}\right)E\left[g^{C_{T}}|S_{T}=a\right]\alpha +E\left[g^{C_{T}}|S_{T}=b\right](1-\alpha )=\prod_{i=a}^{\omega }{\;f_{i}(g)}^{S_{i,0}},$$

where *a*_*i*_ denotes the *i*th element of a=[A,B,…,Ω].

We find the fixation probability and CCFs from equation (2.2) by inserting certain values of the free variable *g* into it [11]. To find the fixation probability *α*, insert *g* = 1:

$$\left(\prod_{i=a}^{\omega }{\;f_{i}(1)}^{a_{i}}\right)\alpha +(1-\alpha )=\prod_{i=a}^{\omega }{\;f_{i}(1)}^{S_{i,0}},$$

and solve for *α*:

$$\alpha =\frac{\prod_{i=a}^{\omega }{\;f_{i}(1)}^{S_{i,0}}-1}{\prod_{i=a}^{\omega }{\;f_{i}(1)}^{a_{i}}-1}.$$

For the CCFs, insert $g=e^{\tau }$ into equation (2.2), where *τ* is a purely imaginary free variable:

$$\left(\prod_{i=a}^{\omega }{\;f_{i}(e^{\tau })}^{a_{i}}\right)E\left[e^{\tau C_{T}}|S_{T}=a\right]\alpha +E\left[e^{\tau C_{T}}|S_{T}=b\right](1-\alpha )=\prod_{i=a}^{\omega }{\;f_{i}(e^{\tau })}^{S_{i,0}}.$$

We identify the conditional expectations as the CCFs of *C*_*T*_, $\psi_{C_{T}|a}(\tau )$ and $\psi_{C_{T}|b}(\tau )$:

$$\left(\prod_{i=a}^{\omega }{\;f_{i}(e^{\tau })}^{a_{i}}\right)\psi_{C_{T}|a}(\tau )\alpha +\psi_{C_{T}|b}(\tau )(1-\alpha )=\prod_{i=a}^{\omega }{\;f_{i}(e^{\tau })}^{S_{i,0}}.$$

Assume that every *f*_*i*_ is convex such that each has two valid complex values in the neighbourhood about *τ* = 0 [30]. Call those values *f*_1,*i*_ and *f*_2,*i*_. Inserting each of those values into equation (2.2) separately, we obtain a system of two equations:

2.3 $$\begin{array}{cc} & \left(\prod_{i=a}^{\omega }{\;f_{1,i}(e^{\tau })}^{a_{i}}\right)\psi_{C_{T}|a}(\tau )\alpha +\psi_{C_{T}|b}(\tau )(1-\alpha )=\prod_{i=a}^{\omega }{\;f_{1,i}(e^{\tau })}^{S_{i,0}}\\ \mathrm{and}\;\;\; & \left(\prod_{i=a}^{\omega }{\;f_{2,i}(e^{\tau })}^{a_{i}}\right)\psi_{C_{T}|a}(\tau )\alpha +\psi_{C_{T}|b}(\tau )(1-\alpha )=\prod_{i=a}^{\omega }{\;f_{2,i}(e^{\tau })}^{S_{i,0}}. \end{array}\}$$

With two equations, we can solve for both $\psi_{C_{T}|a}(\tau )$ and $\psi_{C_{T}|b}(\tau )$.

To apply this analysis, we need to meet one key condition:

2.4 $$E\left[g^{Y_{t}}\prod_{i=a}^{\omega }{\;f_{i}(g)}^{X_{i,t}}|S_{t-1},C_{t-1}\right]=1,$$

for some convex, *state-independent* functions *f*_*i*_(*g*). We now show that the Moran process on a *k*-partite street graph can meet this condition.

### 2.3. A three-dimensional martingale for the tripartite street graph

For simplicity, we will derive *α*, $\psi_{C_{T}|a}(\tau )$ and $\psi_{C_{T}|b}(\tau )$ for a tripartite street graph, e.g. the graph illustrated in figure 1. Later we will generalize the approach for street graphs with any number of partitions.

To condense our notation, let $F_{t-1}$ represent the total fitness of the tripartite street graph on time step *t* − 1:

$$F_{t-1}=rS_{a,t-1}+A-S_{a,t-1}+rS_{b,t-1}+B-S_{b,t-1}+rS_{c,t-1}+C-S_{c,t-1}.$$

We also use compact notation for the graph’s transition probabilities:

$$\begin{array}{rl} & \;p_{X_{a}↑}=\mathrm{Pr}(X_{t}=[1,0,0],Y_{t}=1|S_{t-1}),\;\;p_{X_{a}↓}=\mathrm{Pr}(X_{t}=[-1,0,0],Y_{t}=1|S_{t-1});\\ & \;p_{X_{b}↑}=\mathrm{Pr}(X_{t}=[0,1,0],Y_{t}=1|S_{t-1}),\;\;p_{X_{b}↓}=\mathrm{Pr}(X_{t}=[0,-1,0],Y_{t}=1|S_{t-1});\\ & \;p_{X_{c}↑}=\mathrm{Pr}(X_{t}=[0,0,1],Y_{t}=1|S_{t-1}),\;\;p_{X_{c}↓}=\mathrm{Pr}(X_{t}=[0,0,-1],Y_{t}=1|S_{t-1});\\ \; & \;p_{X0}=\mathrm{Pr}(X_{t}=[0,0,0],Y_{t}=0|S_{t-1}). \end{array}$$

These transition probabilities are independent of *C*_*t*−1_. For the tripartite street graph in figure 1, they are:

$$\begin{array}{rl} & \;p_{X_{a}↑}=\frac{rS_{c,t-1}}{F_{t-1}}\frac{A-S_{a,t-1}}{A},\;\;p_{X_{a}↓}=\frac{B-S_{b,t-1}}{F_{t-1}}\frac{S_{a,t-1}}{A};\\ & \;p_{X_{b}↑}=\frac{rS_{a,t-1}}{F_{t-1}}\frac{B-S_{b,t-1}}{B},\;\;p_{X_{b}↓}=\frac{C-S_{c,t-1}}{F_{t-1}}\frac{S_{b,t-1}}{B};\\ & \;p_{X_{c}↑}=\frac{rS_{b,t-1}}{F_{t-1}}\frac{C-S_{c,t-1}}{C},\;\;p_{X_{c}↓}=\frac{A-S_{a,t-1}}{F_{t-1}}\frac{S_{c,t-1}}{C};\\ \mathrm{and}\;\;\;\;\; & \;p_{X0}=1-\;p_{X_{a}↑}-\;p_{X_{b}↑}-\;p_{X_{c}↑}-\;p_{X_{a}↓}-\;p_{X_{b}↓}-\;p_{X_{c}↓}. \end{array}$$

Our goal is to find state-independent functions *f*_*a*_(*g*), *f*_*b*_(*g*) and *f*_*c*_(*g*) that make equation (2.4) true. The expectation in equation (2.4) is:

$$\begin{array}{rl} E\left[g^{Y_{t}}\prod_{i=a}^{c}{\;f_{i}(g)}^{X_{i,t}}|S_{t-1},C_{t-1}\right] & =\;p_{X_{a}↑}gf_{a}(g)+\;p_{X_{a}↓}gf_{a}{\left(g\right)}^{-1}+\;p_{X_{b}↑}gf_{b}(g)\\ & \;+\;p_{X_{b}↓}gf_{b}{\left(g\right)}^{-1}+\;p_{X_{c}↑}gf_{c}(g)+\;p_{X_{c}↓}gf_{c}{\left(g\right)}^{-1}+\;p_{X0}=1. \end{array}$$

Next, we insert *p*_*X*0_ and rearrange:

2.5 $$\begin{array}{rl} & \;p_{X_{a}↑}gf_{a}(g)+\;p_{X_{a}↓}gf_{a}{\left(g\right)}^{-1}+\;p_{X_{b}↑}gf_{b}(g)+\;p_{X_{b}↓}gf_{b}{\left(g\right)}^{-1}+\;p_{X_{c}↑}gf_{c}(g)+\;p_{X_{c}↓}gf_{c}{\left(g\right)}^{-1}\\ & =\;p_{X_{a}↑}+\;p_{X_{a}↓}+\;p_{X_{b}↑}+\;p_{X_{b}↓}+\;p_{X_{c}↑}+\;p_{X_{c}↓}. \end{array}$$

Equation (2.5) is true when the following three equations are true:

$$\begin{array}{rl} & \;p_{X_{a}↑}gf_{a}(g)+\;p_{X_{c}↓}gf_{c}{\left(g\right)}^{-1}=\;p_{X_{a}↑}+\;p_{X_{c}↓},\;\\ & \;p_{X_{b}↑}gf_{b}(g)+\;p_{X_{a}↓}gf_{a}{\left(g\right)}^{-1}=\;p_{X_{b}↑}+\;p_{X_{a}↓}\\ \mathrm{and}\;\;\;\;\;\; & \;p_{X_{c}↑}gf_{c}(g)+\;p_{X_{b}↓}gf_{b}{\left(g\right)}^{-1}=\;p_{X_{c}↑}+\;p_{X_{b}↓}. \end{array}$$

When we split equation (2.5) like this, we can cancel all state dependence in the transition probabilities:

$$\begin{array}{rl} & \frac{r}{A}gf_{a}(g)+\frac{1}{C}gf_{c}{\left(g\right)}^{-1}=\frac{r}{A}+\frac{1}{C},\;\\ & \frac{r}{B}gf_{b}(g)+\frac{1}{A}gf_{a}{\left(g\right)}^{-1}=\frac{r}{B}+\frac{1}{A}\\ \mathrm{and}\;\;\;\;\;\; & \frac{r}{C}gf_{c}(g)+\frac{1}{B}gf_{b}{\left(g\right)}^{-1}=\frac{r}{C}+\frac{1}{B}. \end{array}$$

With three equations, we can solve for three *state-independent* unknowns *f*_*a*_(*g*), *f*_*b*_(*g*) and *f*_*c*_(*g*) as functions of *g*. These three equations are non-degenerate hyperbolas. So we can find two complex solutions (*f*_1,*a*_, *f*_1,*b*_, *f*_1,*c*_) and (*f*_2,*a*_, *f*_2,*b*_, *f*_2,*c*_), both functions of *g*, that satisfy this system of three equations. Obtaining those solutions requires laborious algebraic manipulation, and their expressions are prohibitively long to state here. We obtained them with the Python package sympy, and our implementation of it may be accessed online at https://github.com/travismonk/kpartite. Since these functions’ expressions are particularly long, sympy requires around 20 seconds to obtain them for the tripartite graph on a standard MacBook Pro laptop. As we increase the number of partitions, sympy requires significantly more time to solve systems with more equations. However, given parameter values of *r* and partition sizes, a numerical solver can solve for these functions much more quickly for a specific graph.

**Figure 2.**
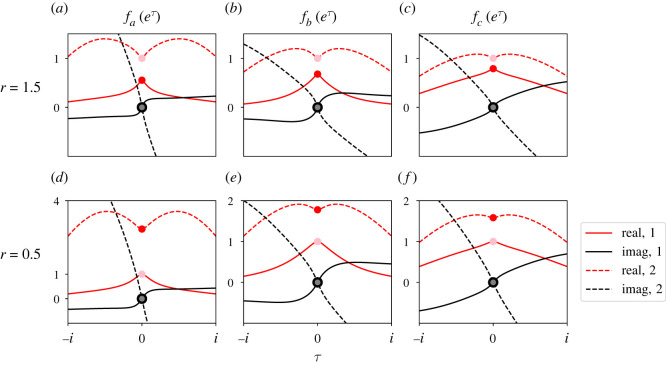
We find two complex solutions to three state-independent functions such that equation (2.4) is true for tripartite street graphs. Therefore, we can find *α*, $\psi_{C_{T}|a}(\tau )$ and $\psi_{C_{T}|b}(\tau )$ immediately. Each panel plots two complex solutions (dashed and solid traces, respectively). Real (red) and imaginary (black) parts of those complex solutions are plotted separately. Each column shows those two solutions for each respective function (column titles). We repeated our analysis for two values of *r* (top and bottom rows). We plot these solutions with respect to *τ*, where $g=e^{\tau }$. Note that the real and imaginary parts are even and odd, respectively. Each solution crosses *g* = 1 (or *τ* = 0) twice. One crossing is trivial at (1, 0*i*) (pink and grey dots), and the other is non-trivial (red, black dots). We use the non-trivial crossing to obtain the fixation probability. In all plots, *A* = 5, *B* = 3 and *C* = 2.

Figure 2 plots both complex solutions of *f*_*a*_(*g*) (left column), *f*_*b*_(*g*) (middle column) and *f*_*c*_(*g*) (right column) as functions of *τ*, where $g=e^{\tau }$. The solid traces plot one solution, and the dashed traces plot the other. The real (red traces) and imaginary (black traces) parts of each solution are plotted separately. We plot them for *r* = 1.5 (top row) and *r* = 0.5 (bottom row). In all plots, we set *A* = 5, *B* = 3 and *C* = 2 like the illustration in figure 1. Given these two solutions, we have found a suitable martingale to immediately extract *α*, $\psi_{C_{T}|a}(\tau )$ and $\psi_{C_{T}|b}(\tau )$.

To obtain the fixation probability *α*, we insert *g* = 1 (or, equivalently, *τ* = 0) into equation (2.2). Figure 2 shows that each function has two valid values at *τ* = 0. The imaginary parts of those values are always zero (black and grey dots). The real part of one of those values is one (pink dots). This value reflects that equation (2.4) has a trivial solution *f*_*a*_ = *f*_*b*_ = *f*_*c*_ = *g* = 1 that we discard. The real part of the other value is non-trivial (red dots, figure 2). Inserting *g* = 1 into our system of three hyperbolas and solving, we find that those non-trivial values have compact expressions:

$$f_{a}(1)=\frac{A+Br^{2}+Cr}{Ar^{3}+Br^{2}+Cr},\;\;f_{b}(1)=\frac{Ar+B+Cr^{2}}{Ar+Br^{3}+Cr^{2}}\;\mathrm{and}\;f_{c}(1)=\frac{Ar^{2}+Br+C}{Ar^{2}+Br+Cr^{3}}.$$

The fixation probability is then:

2.6 $$\alpha =\frac{\;f_{a}{\left(1\right)}^{S_{a,0}}f_{b}{\left(1\right)}^{S_{b,0}}f_{c}{\left(1\right)}^{S_{c,0}}-1}{f_{a}{\left(1\right)}^{A}f_{b}{\left(1\right)}^{B}f_{c}{\left(1\right)}^{C}-1},$$

which verifies previous results [26]. This expression for the fixation probability is undefined when *r* = 1 (i.e. neutral selection), but we can take its limit as *r* → 1:

$$\lim_{r\to 1}\alpha =\frac{AS_{a,0}+BS_{b,0}+CS_{c,0}}{A^{2}+B^{2}+C^{2}}.$$

To find the CCFs of *C*_*T*_, we rearrange equations (2.3):

2.7 $$\psi_{C_{T}|a}(\tau )=\frac{\prod_{i=a}^{c}{\;f_{1,i}}^{S_{i,0}}-\prod_{i=a}^{c}{\;f_{2,i}}^{S_{i,0}}}{\alpha \left(\prod_{i=a}^{c}{\;f_{1,i}}^{a_{i}}-\prod_{i=a}^{c}{\;f_{2,i}}^{a_{i}}\right)}\;\mathrm{and}\;\psi_{C_{T}|b}(\tau )=\frac{\prod_{i=a}^{c}{\;f_{1,i}}^{a_{i}}{\;f_{2,i}}^{S_{i,0}}-\prod_{i=a}^{c}{\;f_{2,i}}^{a_{i}}{\;f_{1,i}}^{S_{i,0}}}{(1-\alpha )\left(\prod_{i=a}^{c}{\;f_{1,i}}^{a_{i}}-\prod_{i=a}^{c}{\;f_{2,i}}^{a_{i}}\right)},$$

and insert our two complex solutions (figure 2) into equations (2.7).

In the special case *A* = *B* = *C* (i.e. the street graph is isothermal), those complex solutions simplify to:

$$f_{1,a}=f_{1,b}=f_{1,c}=\frac{(r+1)\;e^{-\tau }+\sqrt{{\left(r+1\right)}^{2}\;e^{-2\tau }-4r}}{2r}\equiv f_{1}$$

and

$$f_{2,a}=f_{2,b}=f_{2,c}=\frac{(r+1)\;e^{-\tau }-\sqrt{{\left(r+1\right)}^{2}\;e^{-2\tau }-4r}}{2r}\equiv f_{2}.$$

The functions *f*_1,*i*_ become equivalent, as do *f*_2,*i*_ for *i* ∈ [*a*, *b*, *c*]. Therefore our three-dimensional product martingale reduces to a single dimension. Moreover, *f*_1_ and *f*_2_ are equivalent to functions that we previously derived for the fully connected (i.e. one dimensional) Moran process (cf. eqn (2.7) in [11]). When the tripartite street graph is isothermal, $\psi_{C_{T}|a}(\tau )$ and $\psi_{C_{T}|b}(\tau )$ are equivalent to those of the Moran process.

### 2.4. Parameter dependence of $\psi_{C_{T}|a}(\tau )$ and $\psi_{C_{T}|b}(\tau )$

The parameter dependence of our expressions for $\psi_{C_{T}|a}(\tau )$ and $\psi_{C_{T}|b}(\tau )$ is explicit. Therefore, we can explore how the CCFs vary over parameter space by simply evaluating them with different parameter values.

#### 2.4.1. $\psi_{C_{T}|a}(\tau )$ and $\psi_{C_{T}|b}(\tau )$ are relatively insensitive to selection

**Figure 3.**
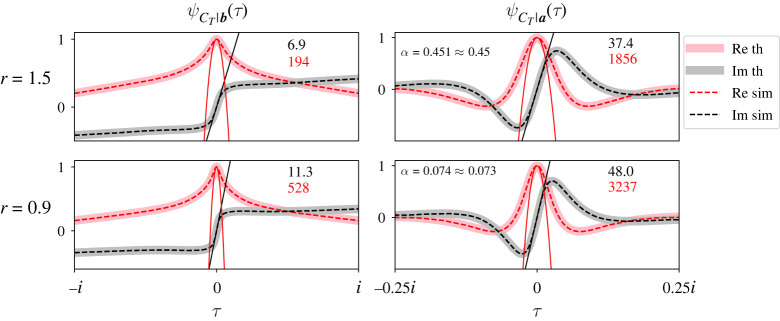
$\psi_{C_{T}|a}(\tau )$ and $\psi_{C_{T}|b}(\tau )$ are somewhat insensitive to changes in *r* for the tripartite street graph in figure 1. Each panel plots the full, exact CCFs of *C*_*T*_ (solid traces) conditional on extinction (left column) or fixation (right column) and compares them with simulations (dashed traces). Real (pink or red) and imaginary (grey or black) parts of the CCFs are plotted separately. We also compare the theoretical fixation probability with the percentage of successful fixations (*α*, right panels). We performed these comparisons for two values of *r* (top and bottom rows). Theory and simulation match very closely because our analysis is exact. The black line and red parabola illustrate how to visualize the first two (conditional) moments of *C*_*T*_. The black and red numbers in each panel report the slope of the line and the concavity of the parabola, respectively (i.e. the values of those first two moments). We see that increasing *r* drastically increases the fixation probability for the graph in figure 1. The CCFs are somewhat affected, but not as drastically. In all plots, ***a*** = [5, 3, 2], ***S*_0_** = [1, 0, 0] and mutants reproduce clockwise.

Figure 3 plots $\psi_{C_{T}|b}(\tau )$ (left column) and $\psi_{C_{T}|a}(\tau )$ (right column) for the tripartite street graph shown in figure 1. We plot the CCFs for two values of *r* (top and bottom rows), with initial mutant population size *S*_0_ = [1, 0, 0] and partition sizes *a* = [5, 3, 2]. The real (pink) and imaginary (grey) parts of the CCFs are plotted separately. Note that the real parts of the CCFs are even and pass through 1 at *τ* = 0, and their imaginary parts are odd and pass through 0 at *τ* = 0.

Figure 3 also compares $\psi_{C_{T}|b}(\tau )$ and $\psi_{C_{T}|a}(\tau )$ (solid traces) with simulation results from 200 000 trials of the Moran process on the same tripartite street graph (dashed traces). On each trial, we counted the number of mutant population size changes before absorption, and stored that count conditional on fixation or extinction. Then we applied the Fourier transform to our stored data to compare it to our theoretical CCFs. Again, we plot the real and imaginary parts of our simulation results separately (dashed red and black traces, figure 3). We also compared our expression for *α* with the percentage of simulations where the mutants fixed (upper-left numbers, right panels). Our simulation code is available online at https://github.com/travismonk/kpartite. Simulation results match our theory extremely closely because our analysis is exact, and we ran sufficiently many simulations to converge to that solution.

Figure 3 shows that the CCFs are relatively insensitive to changes in *r* (cf. top and bottom rows). The main difference between the top and bottom rows is that the CCFs are slightly more compacted about *τ* = 0. The first two moments of *C*_*T*_ clarify this observation. We find the conditional *k*th moments of *C*_*T*_ by evaluating derivatives of the CCFs:

$$E\left[C_{T}^{k}|S_{T}=a\right]=i^{-k}\frac{d^{k}}{d\tau^{k}}\psi_{C_{T}|a}(\tau ){|}_{\tau =0}\;\mathrm{and}\;\;E\left[C_{T}^{k}|S_{T}=b\right]=i^{-k}\frac{d^{k}}{d\tau^{k}}\psi_{C_{T}|b}(\tau ){|}_{\tau =0}.$$

For example, the conditional means of *C*_*T*_ are the slope of the tangent of the CCFs’ imaginary parts at *τ* = 0. Figure 3 plots those tangents (thin solid black lines) and reports their slopes (upper-right black numbers in all panels). The conditional second moments of *C*_*T*_ are the concavity of the parabolas fitted to the CCFs’ real parts at *τ* = 0. Figure 3 also plots those parabolas (thin solid red traces) and reports their concavities (upper-right red numbers in all panels). Figure 3 suggests that, as *r* increases beyond weak selection, the CCFs dilate about *τ* = 0. In particular, the conditional first and second moments of *C*_*T*_ decrease. This result suggests that strong selection decreases the number of mutant population size changes before fixation or extinction.

While strong selection expedites fixation and extinction, *C*_*T*_|***a*** and *C*_*T*_|***b*** are not particularly sensitive to changes in *r*. Figure 3 shows that increasing *r* from 0.9 to 1.5 leads to marginal decreases in the first two conditional moments of *C*_*T*_. Higher-order conditional moments of *C*_*T*_ appear to be relatively unaffected as well. But the fixation probability *α* sextuples for these two values of *r*. This result shows that different statistical quantities can have different sensitivities to changes in *r* for street graphs. Since *r* strongly impacts *α* but weakly impacts *C*_*T*_|***a*** and *C*_*T*_|***b***, street graphs strongly amplify the rate of evolution [44].

**Figure 4.**
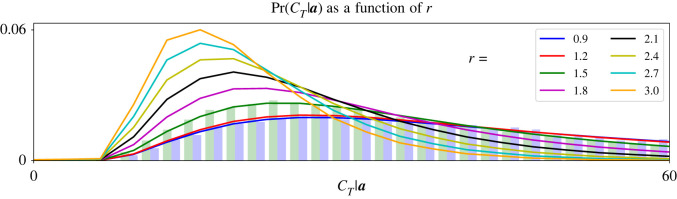
The probability distribution of *C*_*T*_ conditional on fixation concentrates in smaller values as *r* increases. We find the probability distribution of *C*_*T*_|***S***_***T***_ = ***a*** by taking the inverse Fourier transform of its CCF. We plot that conditional distribution for eight values of *r* (legend). Two of those distributions (blue and green traces) are compared with simulation data (blue and green histograms), as we did in figure 3. Our theoretical distributions and simulation histograms match very closely because our analysis is exact. As *r* increases, the mutant population size becomes more likely to require fewer changes before fixing. Therefore, the mean and variance of *C*_*T*_|***S***_***T***_ = ***a*** decrease, as indicated in figure 3 (black and red numbers, right panels, figure 3). In all plots, ***a*** = [5, 3, 2], ***S***_**0**_ = [1, 0, 0] and mutants reproduce clockwise.

Figure 4 plots the probability distribution $\mathrm{Pr}{\left(C_{T}|S_{T}=a\right)}_{t=0}^{\infty }$ of the tripartite graph in figure 1 for various values of *r* (legend). We find the probability distribution of a random variable from its characteristic function via the inverse Fourier transform:

$$\mathrm{Pr}{\left(C_{T}|S_{T}=a\right)}_{t=0}^{\infty }=\frac{1}{2\pi }\int \;e^{-\tau C_{T}}\psi_{C_{T}|a}(\tau )\;d\tau .$$

So figure 4 shows the same results as the right column of figure 3, but as conditional probability distributions instead of CCFs. For example, our simulated CCFs in figure 3 (dashed traces, right column) are represented as histograms in figure 4 (blue and green bars). Those histograms match the probability distributions corresponding to *r* = 0.9 and *r* = 1.5 (blue and green traces, figure 4). Figure 4 shows that as *r* increases, the distribution of *C*_*T*_|***S***_***T***_ = ***a*** loses mass at larger values and concentrates in smaller values. Therefore, its first and second moments decrease as *r* increases, as suggested by figure 3 (red and black numbers, right column, figure 3).

#### 2.4.2. $\psi_{C_{T}|b}(\tau )$ is sensitive to directionality of connections, and $\psi_{C_{T}|a}(\tau )$ is not

We can easily extend our analysis to obtain $\psi_{C_{T}|a}(\tau )$ and $\psi_{C_{T}|b}(\tau )$ for the tripartite street graph where mutants reproduce counter-clockwise and residents clockwise around the graph. A sympy implementation of that analysis is available online at https://github.com/travismonk/kpartite.

**Figure 5.**
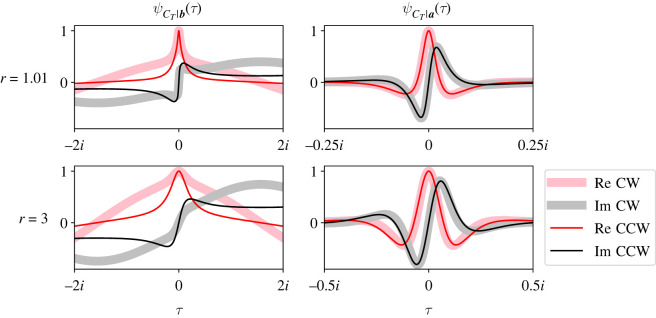
$\psi_{C_{T}|b}(\tau )$ is very sensitive to the directionality of street connections, but $\psi_{C_{T}|a}(\tau )$ is not. Layout is directly analogous to figure 3. Each panel compares our analytical CCFs for a tripartite graph where mutants reproduce clockwise (thick traces) or counter-clockwise (thin traces) around the graph. The directionality of street connections can strongly bias *C*_*T*_|***b***, particularly its higher-order moments (left column). That bias is stronger when selection is strong (cf. top-left and bottom-left panels). Increasing selection marginally expedites fixation (cf. *x*-axes, top-right and bottom-right panels) as it did in figure 3. But the moments of *C*_*T*_|***a*** seem unaffected by connection directionality, regardless of selection. In all plots, ***a*** = [5, 3, 1] and ***S***_**0**_ = [1, 0, 0].

Figure 5 plots $\psi_{C_{T}|a}(\tau )$ (left column) and $\psi_{C_{T}|b}(\tau )$ (right column) for two different values of *r* (top and bottom rows). Again, the real (red or pink traces) and imaginary (black or grey traces) parts of the CCFs are plotted separately. Each panel of figure 5 compares the CCFs when mutants reproduce clockwise around the graph and residents counter-clockwise (thick traces) or vice versa (thin traces). Our partition sizes were ***a*** = [5, 3, 1] and our starting state was ***S***_**0**_ = [1, 0, 0].

The right column of figure 5 shows that $\psi_{C_{T}|a}(\tau )$ is insensitive to the directionality of mutant and resident reproduction around the graph. The right column also reaffirms our results from figure 3 because large increases in *r* do not qualitatively change the CCFs, but dilate them from *τ* = 0 (cf. *x*-axes, right column). Conversely, the left column of figure 5 shows that $\psi_{C_{T}|b}(\tau )$ is very sensitive to the directionality of mutant and resident reproduction. In particular, both left panels show that the $\psi_{C_{T}|b}(\tau )$ noticeably differ from each other far away from *τ* = 0. This divergence indicates that higher-order moments of *C*_*T*_|***S***_***T***_ = ***b*** can strongly depend on the directionality of the street graph’s connections.

To explain this observation, consider a tripartite street graph with ***a*** = [5, 3, 1], ***S***_**0**_ = [1, 0, 0] and let residents reproduce counter-clockwise around the graph. The mutants will go extinct after one population size change if any of the three residents in partition B replace the lonely mutant in partition A. Next consider the same graph, but let the residents reproduce clockwise around the graph. Now the mutants will go extinct after one population size change if the single resident in partition C replaces the lonely mutant in partition A. The probability of the former graph going extinct after a single population size change is thrice that of the latter graph. This bias towards quick extinctions in one graph and delayed extinctions in the other can significantly impact higher-order moments of *C*_*T*_|***b***, at least when ***S***_**0**_ is small.

Comparing the top and bottom rows of figure 5, we see that this bias is amplified when selection is strong. The discrepancy between the directionality of the connections also biases toward quicker invasions in one graph with respect to the other. While we see that the $\psi_{C_{T}|a}(\tau )$ in each of the right panels in figure 5 slightly differ from each other, that discrepancy appears minimal. In both right panels, the traces almost overlap.

#### 2.4.3. Asymmetric partition sizes delay fixation, but extinction is more complicated

**Figure 6.**
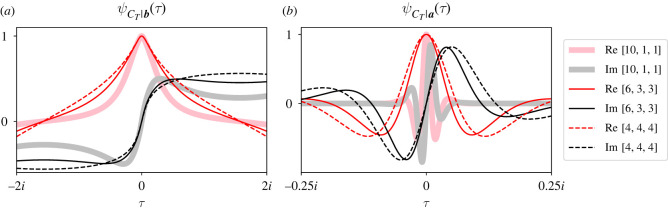
Distributing individuals asymmetrically between three partitions impacts $\psi_{C_{T}|b}(\tau )$ and $\psi_{C_{T}|a}(\tau )$. Each panel compares the extinction (*a*) and fixation (*b*) CCFs of *C*_*T*_ for three tripartite graphs with 12 total individuals (legend). One graph is isothermal (dashed traces), one is somewhat asymmetric (solid thin traces) and one has almost all individuals in one partition (solid thick traces). Real (pink or red) and imaginary (grey or black) parts of the CCFs are plotted separately. Asymmetric partition sizes condense both CCFs about *τ* = 0, particularly for *C*_*T*_|***a***. Therefore, asymmetric partition sizes increase the conditional moments of *C*_*T*_, at least when *r* = 2, ***S***_**0**_ = [0, 1, 0], and mutants reproduce clockwise.

Figure 6 plots three CCFs of $\psi_{C_{T}|b}(\tau )$ (figure 6*a*) and $\psi_{C_{T}|a}(\tau )$ (figure 6*b*). Again, the real (pink or red) and imaginary (grey or black) parts of the CCFs are plotted separately. Each CCF corresponds to one tripartite street graph where mutants reproduce clockwise and residents counter-clockwise around the graph. For each graph, we set *r* = 2 and ***S***_**0**_ = [0, 1, 0], but each graph has different partition sizes (figure 6, legend). Specifically, we fixed the total population size to 12, but we varied how many of those individuals were located in each partition.

Figure 6 shows that asymmetric partition sizes delay fixation and extinction for these parameter values. We see that the graph with the most asymmetric partition sizes (i.e. ***a*** = [10, 1, 1]) has the largest first and second moments in both panels. Conversely, the isothermal graph (i.e. ***a*** = [4, 4, 4]) has the smallest first and second moments for each CCF. We can explain this observation. When ***a*** = [10, 1, 1], ***S***_**0**_ = [0, 1, 0] and residents reproduce counter-clockwise, *C*_*T*_|***b*** = 1 when the resident in partition C reproduces. But when ***a*** = [4, 4, 4], *C*_*T*_|***b*** = 1 when any of the four residents in partition C reproduce. Since such quick extinctions are more likely for the isothermal graph, we expect the moments of $\psi_{C_{T}|b}(\tau )$ to be smaller than for graphs with asymmetric partition sizes.

We also expect asymmetric partition sizes to delay fixation. When the invasion fixes, the mutant in partition B must replace the resident in partition C on the first population size change, i.e. $X_{1}|(Y_{1}=1)=[0,0,1]$. Then we have two possibilities for the second population size change: either ***X***_2_|(*Y*_2_ = 1) = [1, 0, 0] or [0, 0, − 1]. When partition A has a large number of residents, it is more likely that one of them is chosen to replace the mutant in partition C than the other way around. In this more likely scenario, the mutant population size has changed twice, and it has returned to where it started. This argument suggests that when partition sizes are asymmetric, most mutant population size changes in successful fixations will be the few individuals in partition C flipping between mutant and resident. But when the graph is isothermal, the possibility that ***X*_2_**|(*Y*_2_ = 1) = [1, 0, 0] is more likely than it was when partition sizes were asymmetric. Therefore, the invasion more easily spreads between partitions and achieves fixation in fewer population size changes. These observations are consistent with previous simulation results showing that the fixation time *T* increases with the asymmetry of partition sizes [25].

**Figure 7.**
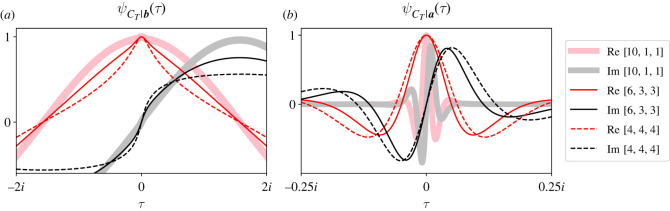
Changing the initial mutant’s location impacts the effect of asymmetric partition sizes on $\psi_{C_{T}|a}(\tau )$. Layout is directly analogous to figure 6. The only difference is that ***S***_**0**_ = [0, 0, 1]. Comparing the right panels of figures 6 and 7, we see that the moments of *C*_*T*_|***a*** are hardly affected by which partition an initial mutant occupies. Comparing their left panels, we see that the moments of *C*_*T*_|***b*** are more affected by both partition size asymmetry and ***S***_**0**_. The probability of observing a quick extinction depends on how many residents are eligible to replace the initial mutant. That number of residents in turn depends on both the asymmetry of partition sizes and the partition occupied by the initial mutant. In these plots, *r* = 2 and mutants reproduce clockwise.

Figure 7 is directly analogous to figure 6, except now we move the initial mutant to partition C, i.e. ***S***_**0**_ = [0, 0, 1]. Note that the CCFs for the isothermal graph (dashed traces) are the same as they were in figure 6. The symmetry of the isothermal graph implies that both its CCFs remain invariant to which partition the initial mutant occupies. However, note that the other two extinction CCFs (thick and thin solid traces, left panel) are dilated about *τ* = 0 with respect to those in figure 6. This dilation implies that the moments of *C*_*T*_|***b*** decrease when partition sizes are asymmetric and we move the initial mutant from partition B to partition C. When the initial mutant occupies partition B, *C*_*T*_|***b*** = 1 only if the resident in C is chosen to reproduce. When we place it in partition C, *C*_*T*_|***b*** = 1 when any resident in A is chosen to reproduce. So when the initial mutant occupies partition C instead of partition B, a quick extinction is more likely.

Comparing $\psi_{C_{T}|a}(\tau )$ (figure 7*b*) with those of figure 6, we see that the fixation CCF is relatively unaffected by changing the partition of the initial mutant. Again we observe that, as partition sizes become asymmetric, $\psi_{C_{T}|a}(\tau )$ condenses about *τ* = 0. Therefore, the moments of *C*_*T*_|***a*** increase when partition sizes are asymmetric, as we observed in figure 6. We also notice that the solid traces in figure 7 are very similar to those in figure 6. If partition sizes are asymmetric, ***S***_**0**_ = [0, 0, 1], and the process fixes, then that first mutant in partition C will reproduce around the graph until one of its descendants occupies partition B. The process will closely resemble our previous starting state ***S***_**0**_ = [0, 1, 0] from figure 6 after a few population size changes at the beginning of the invasion. So we would expect the fixation CCFs for the two starting states to be almost identical, excluding the first few ‘active steps’.

#### 2.4.4. $\psi_{C_{T}|a}(\tau )$ is more sensitive to graph dimensionality than $\psi_{C_{T}|b}(\tau )$

Next, we extend our results from a tripartite street graph to a street graph with five partitions (so *k* = 5 instead of 3, and *ω* = *e* instead of *c*). In doing so it should be obvious how martingale methodology applies to street graphs with any number of partitions.

For a street graph with five partitions, our key condition to apply martingale analysis is:

$$E\left[g^{Y_{t}}\prod_{i=a}^{e}{\;f_{i}(g)}^{X_{i,t}}|S_{t-1},C_{t-1}\right]=1,$$

where we define five *state-independent* functions *f*_*i*_(*g*), *i* ∈ [*a*, *e*], one for each graph partition. Writing the expectation:

$$\sum_{i=a}^{e}\;p_{X_{i}↑}gf_{i}(g)+\sum_{i=a}^{e}\;p_{X_{i}↓}g{\;f_{i}(g)}^{-1}+1-\sum_{i=a}^{e}\;p_{X_{i}↑}-\sum_{i=a}^{e}\;p_{X_{i}↓}=1,$$

where $\;p_{X_{i}↑}$ and $\;p_{X_{i}↓}$ are the transition probabilities of the graph. We wrote *p*_*X*0_ as 1 minus two sums so we can rearrange our key condition:

2.8 $$\sum_{i=a}^{e}\;p_{X_{i}↑}gf_{i}(g)+\sum_{i=a}^{e}\;p_{X_{i}↓}g{\;f_{i}(g)}^{-1}=\sum_{i=a}^{e}\;p_{X_{i}↑}+\sum_{i=a}^{e}\;p_{X_{i}↓}.$$

Next, we want to cancel state dependence from the 10 transition probabilities. Note that the state dependence of mutants reproducing into one partition matches the state dependence of residents reproducing in the opposite direction. For example, consider a street graph where mutants reproduce clockwise around the graph. Two transition probabilities are:

$$\;p_{X_{b}↑}=\frac{rS_{a,t-1}}{F_{t-1}}\frac{B-S_{b,t-1}}{B}\;\mathrm{and}\;\;p_{X_{a}↓}=\frac{B-S_{b,t-1}}{F_{t-1}}\frac{S_{a,t-1}}{A}.$$

The state dependence in both transition probabilities is *S*_*a*,*t*−1_(*B* − *S*_*b*,*t*−1_). Every transition probability can be paired with another that has identical state dependence. Equation (2.8) has 10 transition probabilities, and we can eliminate all state dependence by splitting it into five equations, each containing one such pair. For example, one of those five equations would be:

$$\;p_{X_{b}↑}gf_{b}(g)+\;p_{X_{a}↓}g{\;f_{a}(g)}^{-1}=\;p_{X_{b}↑}+\;p_{X_{a}↓}$$

and we can cancel *S*_*a*,*t*−1_(*B* − *S*_*b*,*t*−1_) in the transition probabilities.

Splitting equation (2.8) into five equations and cancelling state dependence results in five *state-independent* equations with five unknowns *f*_*i*_(*g*), *i* ∈ [*a*, *e*] to find. All five equations are non-degenerate hyperbolas, so we can find two complex solutions that solve the whole system. Unfortunately, obtaining analytical expressions for those solutions is algebraically tedious and their forms are not compact. We used sympy to find *f*_*i*_(*g*), *i* ∈ [*a*, *e*] for a street graph with five partitions (visit https://github.com/travismonk/kpartite). Given those two complex solutions, we can immediately calculate *α*, $\psi_{C_{T}|a}(\tau )$ and $\psi_{C_{T}|b}(\tau )$.

Martingale methodology is quickly generalized to any number of partitions in a street graph. Given *k* partitions, there are 2*k* transition probabilities. Each transition probability can be paired with another that has the same state dependence. When we evaluate equation (2.4), we can split it into a system of *k* equations such that state dependence cancels in all of them. Each state-independent equation will be a non-degenerate hyperbola. So we can find two complex solutions that satisfy the system and directly obtain *α*, $\psi_{C_{T}|a}(\tau )$ and $\psi_{C_{T}|b}(\tau )$.

**Figure 8.**
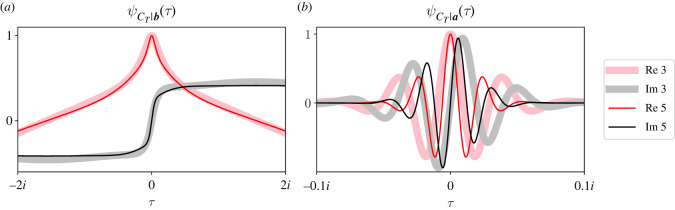
Increasing the number of partitions delays fixation more than extinction. Each panel compares the extinction (*a*) and fixation (*b*) CCFs of *C*_*T*_ for a street graph with three (thick solid traces) or five (thin solid traces) partitions. For the tripartite graph, ***a*** = [24, 12, 12], and for the five-partition graph, ***a*** = [24, 6, 6, 6, 6], so both graphs have the same total number of individuals. Increasing the number of partitions delays extinction when fewer residents can replace an initial mutant on the first population size change (*a*). However, the extinction CCF seems relatively unaffected by the extra partitions. The fixation CCF (*b*) is more noticeably affected by adding extra partitions. By spreading half the total population among twice as many partitions, we increase the asymmetry in partition sizes. So we expect the five-partition graph to achieve fixation after more mutant population size changes, as observed in figures 6 and 7. In these plots, *r* = 2, ***S***_**0**_ = [1, 0, 0] or [1, 0, 0, 0, 0] and mutants reproduced clockwise.

Figure 8 compares $\psi_{C_{T}|b}(\tau )$ (figure 8*a*) and $\psi_{C_{T}|a}(\tau )$ (figure 8*b*) for street graphs with three (thick traces) or five (thin traces) partitions. Again the real (pink or red) and imaginary (grey or black) parts of the CCFs are plotted separately. Both street graphs have 48 total individuals with partition sizes ***a*** = [24, 12, 12] or [24, 6, 6, 6, 6]. In both graphs, mutants reproduced clockwise, we set *r* = 2, and our starting state was one mutant in partition A.

Figure 8*a* shows that the general form of $\psi_{C_{T}|b}(\tau )$ is relatively insensitive to the number of partitions in the street graph. When selection is strong, we expect the moments of *C*_*T*_|***b*** to be small (recall figure 3, upper-left panel), regardless of the graph’s dimensionality. Closer inspection about *τ* = 0 shows that the first two moments of *C*_*T*_|***b*** for the five-partition graph are higher than those of the tripartite graph. For the five-partition graph, the initial mutant in partition A is half as likely to die upon the first population size change, because it has half as many residents able to replace it. So we expect the first two moments of *C*_*T*_|***b*** for the five-partition graph to be larger, as shown in figure 8*a*.

Figure 8*b* shows that adding additional partitions condenses $\psi_{C_{T}|a}(\tau )$ about the origin. Therefore, adding more partitions increases the moments of *C*_*T*_|***a***. We expect this result because the partition sizes are more asymmetric in the five-partition graph than they are in the tripartite graph. Figures 6*b* and 7*b* show that asymmetric partition sizes delay fixation. Figure 8 is consistent with those results.

### 2.5. Extension to more general street graphs

Our martingale methodology can be applied to street graphs beyond the cyclically connected examples we have considered so far.

**Figure 9.**
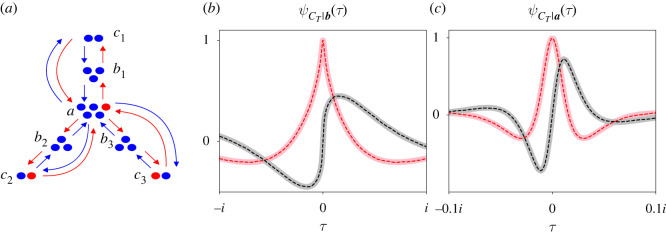
Our martingale analysis generalizes to consider a megastar street graph, where each leaf is a *k*-partite street graph. It is particularly easy to extend our analysis when the megastar’s leaves are identical. (*a*) An example megastar street graph with three identical leaves. The leaves overlap at one central partition. Mutant offspring travel away from that central partition and toward the ends of the leaves, and resident offspring travel in the other direction. (*b*) We compare our theoretical CCF of *C*_*T*_ conditional on extinction (solid pink and grey traces) with simulation results over 100 000 trials (dashed red and black traces). (*c*) We show an analogous comparison for the CCF of *C*_*T*_ conditional on fixation. In these plots, *r* = 1.2, and the partition sizes and ***S***_**0**_ are illustrated in (*a*).

Figure 9*a* illustrates an example of a street graph whose partitions are not cyclically connected, but is still amenable to our analysis. This example resembles a megastar graph [5,37], but with street connections instead of directed connections [26]. Each ‘leaf’ of the megastar is identical to the tripartite street graph introduced in figure 1. Mutant offspring travel towards the tips of the leaves, and then back to the centre partition (red arrows). Resident offspring travel in the opposite direction (blue arrows). We create an alphabet to index the partitions as shown in figure 9.

This megastar street graph has seven partitions and 14 transition probabilities:

$$\begin{array}{rl} & \;p_{X_{a}↑}=\sum_{\;j=1}^{3}\frac{rS_{c,j,t-1}}{F_{t-1}}\frac{A-S_{a,t-1}}{A};\;\;p_{X_{a}↓}=\sum_{\;j=1}^{3}\frac{B_{j}-S_{b,j,t-1}}{F_{t-1}}\frac{S_{a,t-1}}{A};\\ & \;p_{X_{b,j}↑}=\frac{rS_{a,t-1}}{F_{t-1}}\frac{B_{j}-S_{b,j,t-1}}{B_{j}};\;\;p_{X_{b,j}↓}=\frac{C_{j}-S_{c,j,t-1}}{F_{t-1}}\frac{S_{b,j,t-1}}{B_{j}};\\ \; & \;p_{X_{c,j}↑}=\frac{rS_{b,j,t-1}}{F_{t-1}}\frac{C_{j}-S_{c,j,t-1}}{C_{j}};\;\;p_{X_{c,j}↓}=\frac{A-S_{a,t-1}}{F_{t-1}}\frac{S_{c,j,t-1}}{C_{j}}, \end{array}$$

where *j* ∈ [1, 2, 3] indexes the three leaves of the megastar.

Our key condition to apply martingale analysis is:

$$E\left[g^{Y_{t}}{\;f_{a}(g)}^{X_{a,t}}\prod_{\;j=1}^{3}{\;f_{b,j}(g)}^{X_{b,j,t}}{\;f_{c,j}(g)}^{X_{c,j,t}}|S_{t-1},C_{t-1}\right]=1,$$

where we define seven functions *f*_*a*_(*g*), *f*_*b*,*j*_(*g*) and *f*_*c*,*j*_(*g*) for *j* ∈ [1, 2, 3], one function for each partition in the megastar graph. Writing the expectation, inserting *p*_*X*0_, and splitting the expectation as before, this condition is met when:

$$\begin{array}{rl} & \;p_{X_{a}↑}gf_{a}(g)+\sum_{\;j=1}^{3}\;p_{X_{c,j}↓}gf_{c,j}{\left(g\right)}^{-1}=\;p_{X_{a}↑}+\sum_{\;j=1}^{3}\;p_{X_{c,j}↓},\;\\ & \sum_{\;j=1}^{3}\;p_{X_{b,j}↑}gf_{b,j}(g)+\;p_{X_{a}↓}gf_{a}{\left(g\right)}^{-1}=\sum_{\;j=1}^{3}\;p_{X_{b,j}↑}+\;p_{X_{a}↓}\\ \mathrm{and}\;\;\;\; & \sum_{\;j=1}^{3}\;p_{X_{c,j}↑}gf_{c,j}(g)+\sum_{\;j=1}^{3}\;p_{X_{b,j}↓}gf_{b,j}{\left(g\right)}^{-1}=\sum_{\;j=1}^{3}\;p_{X_{c,j}↑}+\sum_{\;j=1}^{3}\;p_{X_{b,j}↓}. \end{array}$$

Since the leaves are symmetric, the partition sizes of the leaves *B*_*j*_ and *C*_*j*_ and the functions *f*_*b*,*j*_(*g*) and *f*_*c*,*j*_(*g*) corresponding to them are independent of *j*. Inserting the transition probabilities and pulling constant terms out of the sums, we see that all state dependence cancels. For example, the first equation above becomes:

$$\begin{array}{rl} & \frac{r}{A}gf_{a}(g)\sum_{\;j=1}^{3}\frac{S_{c,t-1}(A-S_{a,t-1})}{F_{t-1}}+\frac{1}{C}gf_{c}{\left(g\right)}^{-1}\sum_{\;j=1}^{3}\frac{S_{c,t-1}(A-S_{a,t-1})}{F_{t-1}}\\ & =\frac{r}{A}\sum_{\;j=1}^{3}\frac{S_{c,t-1}(A-S_{a,t-1})}{F_{t-1}}+\frac{1}{C}\sum_{\;j=1}^{3}\frac{S_{c,t-1}(A-S_{a,t-1})}{F_{t-1}}, \end{array}$$

and we can cancel the sums with the state-dependent terms. After cancelling those terms, our key condition is met when the following equations are true:

$$\begin{array}{rl} & \frac{r}{A}gf_{a}(g)+\frac{1}{C}gf_{c}{\left(g\right)}^{-1}=\frac{r}{A}+\frac{1}{C},\;\\ & \frac{r}{B}gf_{b}(g)+\frac{1}{A}gf_{a}{\left(g\right)}^{-1}=\frac{r}{B}+\frac{1}{A}\\ \mathrm{and}\;\;\;\;\;\;\;\; & \frac{r}{C}gf_{c}(g)+\frac{1}{B}gf_{b}{\left(g\right)}^{-1}=\frac{r}{C}+\frac{1}{B}. \end{array}$$

We recognize these equations as identical to those we derived for the tripartite street graph in figure 1. Therefore, the functions *f*_*a*_(*g*), *f*_*b*_(*g*) and *f*_*c*_(*g*) are given by figure 2. So to obtain the fixation probability and CCFs of *C*_*T*_, we simply insert the absorbing states ***a*** = [*A*, 3*B*, 3*C*] and ***b*** = [0, 0, 0] into equations (2.6) and (2.7).

Figure 9*b*,*c* plots the CCFs of *C*_*T*_ for the megastar graph in figure 9*a*. Again, we plot the real (solid pink) and imaginary (solid grey) parts of the CCFs separately. We set *r* = 1.2 and ***S***_**0**_ is indicated by the red dots in figure 9*a*. We compared our theoretical CCFs with simulation results from 100 000 trials of the Moran process on the megastar graph (dashed red and black traces). Simulation results match our theoretical CCFs very closely because our derivation is exact.

Our analysis quickly generalizes to megastar street graphs with any number of leaves *m* as long as the leaves are identical. We find the functions *f*_*i*_(*g*), *i* ∈ [*a*, *b*, …, *ω*] for a cyclical street graph with any number of partitions, as we have already shown. Then we simply insert the absorbing states a=[A,mB,…,mΩ] and b=[0,0,…,0] into our expressions for the fixation probability and CCFs of *C*_*T*_.

### 2.6. Approximating conditional characteristic functions of fixation time

**Figure 10.**
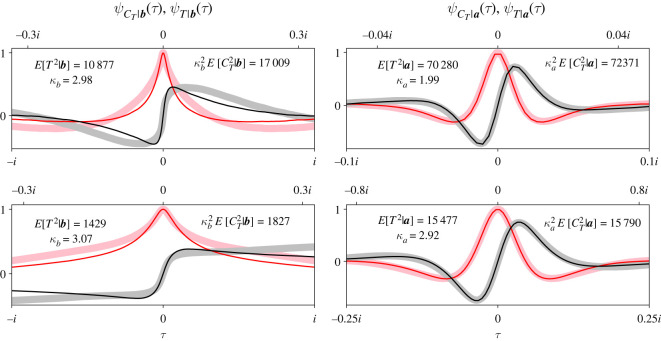
The CCFs of *T* are closely approximated by scaling the CCFs of *C*_*T*_ under certain conditions. We compare the CCFs of *T* (thin red and black traces) with those of *C*_*T*_ (thick pink and grey traces) for the megastar street graph (top row) and tripartite street graph (bottom row). The CCFs are plotted with respect to different scales of *τ* as indicated by the top (*T*) and bottom (*C*_*T*_) *x*-axes of all panels. We obtained the CCFs of *T* by taking the Fourier transform of 100 000 (megastar) or 200 000 (tripartite) simulation results of the Moran process on those graphs. For the megastar graph, we set *r* = 1.2 and ***S***_**0**_ = [1, 0, 2]. For the tripartite graph, we set *r* = 1.5 and ***S***_**0**_ = [1, 0, 0]. When ***S***_**0**_ is small, the CCF of *T*|***a*** is closely approximated by scaling the CCF of *C*_*T*_|***a*** with an appropriate constant *κ*_*a*_ (right column). *T*|***b*** is less accurately approximated by scaling *C*_*T*_|***b*** (left column), particularly for higher-order moments.

Figure 10 compares the CCFs of *C*_*T*_ (thick traces) with simulation results of the CCFs of fixation time *T* (thin traces). Again, we plot the real (pink and red) and imaginary (grey and black) parts of the CCFs separately. The top row contains the CCFs of the megastar graph in figure 9 and the bottom row contains those of the tripartite street graph in figure 1. We used parameter values *r* = 1.2 and ***S***_**0**_ = [1, 0, 2] for the megastar graph (figure 9), and *r* = 1.5 and ***S***_**0**_ = [1, 0, 0] for the tripartite street graph. The left column plots the extinction CCFs and the right column plots the fixation CCFs. In all panels, the CCFs of *C*_*T*_ and *T* are plotted with respect to different scales of *τ*, i.e. different *x*-axes. The CCFs of *C*_*T*_ and *T* are plotted with respect to the *τ* axis at the bottom and top of each panel, respectively.

By comparing scaled versions of the CCFs, we are implicitly approximating that *T*|***a*** ∝ *C*_*T*_|***a*** and *T*|***b*** ∝ *C*_*T*_|***b***. When we scale the independent variable of a CCF, we scale its random variable:

$$\psi_{C_{T}|b}(\kappa_{b}\tau )=\psi_{\kappa_{b}C_{T}|b}(\tau )\approx \psi_{T|b}(\tau )\;\mathrm{and}\;\;\psi_{C_{T}|a}(\kappa_{a}\tau )=\psi_{\kappa_{a}C_{T}|a}(\tau )\approx \psi_{T|a}(\tau ),$$

where *κ*_*b*_ and *κ*_*a*_ are the scaling constants. Figure 10 illustrates the accuracy of this proportionality approximation.

Intuitively, our approximations *T*|***a*** ≈ *κ*_*a*_ *C*_*T*_|***a*** and *T*|***b*** ≈ *κ*_*b*_ *C*_*T*_|***b*** are sensible. The time that the Moran process spends before absorption is the number of times that the mutant population size changes (i.e. *C*_*T*_), multiplied by the amount of time that it remains stuck in each state. But the (geometrically distributed) amount of time that the process spends in each transient state is not identical over all state space. In other words, the sojourn times [24,45] of the Moran process are not constant over its transient states [11]. When the mutant population size is very large or very small, the Moran process can remain in those states for a large number of time steps [10]. When the numbers of mutants and residents are approximately equal, the Moran process changes states more frequently. This disparity is largely offset by the number of times that the Moran process visits its transient states. Transient states close to fixation or extinction barriers are rarely visited, while states far from those barriers are visited frequently. If the time spent per visit in transient states was perfectly cancelled by the number of visits to those states, then the CCFs of *T* would be much simpler to calculate.

While the CCFs of *T* for the Moran process remain an open problem [10,11], figure 10 shows that we can accurately approximate them from the CCFs of *C*_*T*_ under certain conditions. The right column of figure 10 shows that the fixation CCF of *T* is very accurately approximated by scaling the fixation CCF of *C*_*T*_ with an appropriate scaling constant *κ*_*a*_. We defined $\kappa_{a}=E[T|a]/E[C_{T}|a]$ and calculated E[T|a] from our simulation results. Our values for *κ*_*a*_ are reported in each panel. Since $E[T|a]=\kappa_{a}E[C_{T}|a]$ and figure 10 plots the CCFs at different relative scales, the slopes of the imaginary parts of the fixation CCFs at *τ* = 0 are the same. Each panel also reports the scaled second moments $E[T^{2}|a]$ and $E[C_{T}^{2}|a]$ and shows that their values are very similar. The strong overlap of the CCFs indicates that properly scaled higher-order moments are similar in value as well. These results suggest that the sojourn CCFs conditional on fixation do not appreciably vary over the transient states of the Moran process on street graphs, at least when ***S***_**0**_ is small. Therefore, our scaling approximation *T*|***a*** ≈ *κ*_*a*_ *C*_*T*_|***a*** is accurate.

The left column of figure 10 shows that our scaling approximation *T*|***b*** ≈ *κ*_*b*_ *C*_*T*_|***b*** is less accurate. When ***S***_**0**_ is small and mutants go extinct, the sojourn CCFs conditional on extinction vary substantially over transient states of the (fully connected) Moran process [11]. Figure 10 suggests that this result holds for the Moran process on street graphs. Comparing the top and bottom panels of the left column, our scaling approximation becomes less accurate as ***S***_**0**_ moves away from the extinction barrier. For the megastar graph (top row, ***S***_**0**_ = [1, 0, 2]), the scaled second moments have a 56% difference, while for the tripartite graph (bottom row, ***S***_**0**_ = [1, 0, 0]) they have a 28% difference. When ***S***_**0**_ is further from the extinction barrier, mutants are more likely to require more population size changes before going extinct. Therefore, the Moran process is more likely to traverse more transient states whose conditional sojourn times vary appreciably, so our scaling approximation loses accuracy.

## 3. Discussion

Martingales are a powerful tool that can address fundamental problems in EGT and other biologically themed stochastic processes [6,11,26,27,41,46–49]. The mutant population on an evolutionary graph may be considered as a multi-dimensional random walk between two absorbing barriers. We might expect that analytic study of evolutionary graphs suffers from a curse of dimensionality. For example, we can construct a Markov matrix of transition probabilities and attempt to calculate fixation probabilities and times from it [4,7,9,22,50–53]. But as we increase the number of partitions, we increase the number of transition probabilities, and that matrix quickly becomes too complicated to manipulate [38,53–56]. Another popular approach to investigating evolutionary graphs is by exhaustive simulation [12,54,57,58]. But as the number of partitions increases, we increase the dimensionality of parameter space. It is infeasible to explore how fixation probabilities and times depend on graph parameters (i.e. partition sizes) when the number of partitions is large. We have shown that, when they are applicable, martingales directly address this curse.

Martingales are conservation statements for absorbing random walks [11,29]. They show that the expectation of some quantity is constant in time throughout a random walk, regardless of its history or state. So if we know that expectation at the beginning of the random walk, then we know it at the end. We can often extract global statistics of interest from that conservation statement [30–32], as we illustrated here. Crucially, the existence of that conservation statement does not necessarily depend on the dimensionality of the random walk [26]. We showed that a *k*-partite street graph yields a *k*-dimensional product martingale, and from that martingale we can extract *α*, $\psi_{C_{T}|a}(\tau )$ and $\psi_{C_{T}|b}(\tau )$. For street graphs, martingales reduce the curse of dimensionality to the simpler task of finding two complex solutions to a system of quartic equations. Once we have that conservation statement, we immediately obtain elegant and exact expressions for global statistics of interest. There is no need to construct and manipulate a matrix of transition probabilities to do so.

Martingale analysis is more amenable to some random variables than it is for others. For example, we found a product martingale that yields the CCFs of *C*_*T*_, but we have not found one that yields the CCFs of the fixation time *T*. To briefly explain why, say we want to find a product martingale of the form (cf. equation (2.1)):

$$E\left[g^{T}\prod_{i=a}^{k}{\;f_{i}(g)}^{S_{i,t}}|S_{t-1},T-1\right]=g^{T-1}\prod_{i=a}^{k}{\;f_{i}(g)}^{S_{i,t-1}}.$$

This equation is true when:

$$E\left[\prod_{i=a}^{k}{\;f_{i}(g)}^{X_{i,t}}|S_{t-1},T-1\right]=\frac{1}{g}.$$

Writing the expectation:

$$\sum_{i=a}^{k}\;p_{X_{i}↑}f_{i}(g)+\sum_{i=a}^{k}\;p_{X_{i}↓}{\;f_{i}(g)}^{-1}+1-\sum_{i=a}^{k}\;p_{X_{i}↑}-\sum_{i=a}^{k}\;p_{X_{i}↓}=\frac{1}{g}.$$

We note that the number 1 on the left-hand side does not cancel with 1/*g* on the right-hand side. So it is harder to cancel the state dependencies of the transition probabilities like we did before. If we cannot cancel state dependencies, then we cannot find a conservation statement for the random walk and extract global statistics from it. A martingale of *T* may still exist for street graphs, but it does not have the same form as equation (2.1).

The EGT literature primarily studies the conditional distributions or moments of *T* [21,23,24,59,60]. We question whether *T* is an ideal quantity to represent the duration of the Moran process on a graph [41]. The graph’s transition probabilities are unaffected if we eliminate time steps where the graph does not change. So the underlying process is indifferent to whether we include those time steps or not. In simulations, including those time steps can dramatically slow computation time, particularly when the mutant population size is very large or very small [10,33]. Including those time steps also impedes analytical study of the Moran process as we have shown. But if we insist on *T* as our definition of the Moran process duration, we can often approximate its conditional distributions from those of *C*_*T*_ anyway [11].

Martingales are sensitive to certain changes in the graph and the birth–death process that runs on it [61,62]. Here, we studied the original Moran process, where we select the reproducing individual before the dying one on a time step, and only the reproducing phase is fitness-dependent [3]. But we can also consider a ‘death–birth process’ where we choose an individual to die before choosing one to reproduce on a time step [63–67]. If the probability of choosing the dying individual is fitness-dependent, and the reproducing individual is chosen randomly, then martingale analysis remains applicable. But if the dying individual is chosen uniformly at random, and the probability of reproducing is fitness-dependent, then it is much more difficult to cancel the state dependencies in the transition probabilities. Our martingale analysis is applicable to both birth–death and death–birth processes, but only when the first phase of their time steps is fitness-dependent.

Martingales are also sensitive to a graph’s connection type. For example, consider a *k*-partite graph with directed connections instead of street connections. Directed connections constrain the offspring of mutants and residents to travel in only one direction between partitions [37,38]. For street graphs, we found a product martingale by observing that the state dependence of the probability that a mutant reproduces in one direction equals that of a resident reproducing in the other direction. Directed connections destroy that symmetry because mutants and residents can only reproduce in the same direction. It is much harder to find a martingale for graphs with directed connections.

We might be able to apply martingale analysis to *k*-partite graphs with undirected connections [13,34–36], where mutants and residents can reproduce in both directions. Such graphs have 2*k* unique state dependence terms in their transition probabilities of the form *S*_*i*,*t*−1_(*J* − *S*_*j*,*t*−1_). To cancel all those state dependent terms, we need to split equation (2.4) into 2*k* separate equations. Then given 2*k* state-independent equations, we need to solve for 2*k* functions *f*_*i*_(*g*) to find a product martingale. So we can find a product martingale with the same form as equation (2.1) for a *k*-partite graph with undirected connections, but its dimensionality will be 2*k*.

Those extra *k* dimensions complicate the extraction of global statistics from the martingale. Each dimension of the martingale corresponds to one random variable in the random walk. For example, in the *k*-partite street graph, each dimension of the martingale corresponds to the mutant population size of one partition. Since we know the two absorbing barriers for all *k* random variables (i.e. ***S***_***T***_ = ***a*** and ***S***_***T***_ = ***b***), we can manipulate the martingale and extract global statistics as we showed. For undirected *k*-partite graphs, our martingale has 2*k* dimensions. Half of those dimensions can again correspond to the mutant population size in each partition. We know that the two absorbing barriers for those *k* random variables are ***a*** and ***b***. But we still need to find *k* more random variables to correspond to the remaining *k* dimensions in the martingale. Furthermore, we need to know the absorbing barriers of those random variables, given that the mutants fixed or went extinct. If we can find *k* more random variables, then we can extend our analysis to consider undirected *k*-partite graphs.

Martingales provide clean and exact expressions for statistics of interest in EGT. Their parameter dependence is explicit, so we can easily explore the dependence of those statistics on parameter values. Martingales do not require simplifying assumptions such as large population size [5,10] or weak selection [38,56,68] to yield tractable results. The drawback of martingales is that seemingly insignificant changes to a birth–death process can complicate their applicability. But when they are applicable, martingales offer clear advantages over other approaches to studying evolutionary graphs, e.g. simulation [12,54,57] and Markov chain [4,7,22,50–53] or state transition-based [4,7,38,50,51] methodologies. So it seems worthwhile to continue expanding the classes of graphs and problems that martingales can address so aesthetically.

## Data Availability

Data and relevant code for this research work are stored in GitHub: https://github.com/travismonk/kpartite and have been archived within the Zenodo respository: https://doi.org/10.5281/zenodo.6390725.
